# Primary plasma cell leukemia: A case report and review of the literature

**DOI:** 10.1002/ccr3.2339

**Published:** 2019-07-30

**Authors:** Sam Ngu, Divya Asti, Gautam Valecha, Nishitha Thumallapally, Manisha Pant, Alexander Bershadskiy

**Affiliations:** ^1^ Staten Island University Hospital Staten Island New York

**Keywords:** daratumumab, Kyle's criteria, multiple myeloma, plasma cell leukemia

## Abstract

Due to the rarity and fulminant nature of the condition, there are limited data driving dialogue for optimal treatment strategies for plasma cell leukemia (PCL). Additionally, the current diagnostic definition of PCL has not been prospectively studied which may result in delays to initiating early aggressive treatment due to underdiagnosis.

## 1 INTRODUCTION

Plasma cell leukemia (PCL) is a rare and aggressive variant of multiple myeloma (MM) with a generally fulminant course. The disease entity arises in 1%‐4% of patients with MM.^1^ Diagnosis is arbitrarily defined by Kyle's criteria as the presence of more than 20% circulating clonal plasma cells or an absolute plasma cell count greater than 2 x 10^9^/L in peripheral blood.^2^ PCL is further classified as primary (pPCL) when the leukemic phase is present at diagnosis or as secondary (sPCL) when there is leukemic transformation of relapsed or refractory MM. Representing up to 60% of cases of PCL, pPCL poses a significant clinical challenge with dismal survival outcomes even when treated with novel chemotherapy agents and autologous stem cell transplantation (AuSCT). We present a rare case of a 76‐year‐old African American woman with pPCL who was treated with early aggressive chemotherapy. While she did not meet Kyle's criteria, morphological, immunophenotypic and immunohistochemical studies, and overall clinical presentation support the diagnosis. She was initially treated with dexamethasone followed by bortezomib, cyclophosphamide, and daratumumab for 3 cycles with good response. Repeat serum protein electrophoresis (SPEP) and peripheral flow cytometry demonstrated no evidence for clonal B‐cell population, abnormal T‐cell population, or increased blast population. This case illustrates the application of daratumumab as part of a novel agent‐based regimen as a first‐line treatment of pPCL to impart a deeper and more rapid clinical response and the need for a less stringent criteria in its diagnosis. Because these criteria have not been prospectively studied, it may underestimate the true incidence of PCL. This may lead to undertreatment of those who fail to meet the current diagnostic criteria. Current literature suggests that overall poor prognosis could even be seen in patients with peripheral plasmacytosis as low as 1%‐2%.^3^, ^4^

### 1.1 Clinical Features

There are distinct clinical features between PCL and MM. Originally, patients with PCL were thought to be at least 10 years younger (53 to 57 yo) than the median age of diagnosis of MM.^5^, ^6^ However, a recent US registry analysis of 291 patients diagnosed between 1973 and 2004 in Surveillance, Epidemiology and End Results (SEER) database showed no significant demographic differences.^7^ Like MM, PCL is more prevalent amongst African Americans and blacks from Africa.^8^ The prognosis of PCL patients treated with conventional chemotherapy is poor even when compared to MM patients with high tumor burden, with a median overall survival (OS) ranging from 2 to 12 months.^7^ Those less than 60 years of age were found to have better OS compared with those comparatively older (7 month vs 4 month); however, the 5‐year mortality was poor in both groups.^7^ Though studies have shown improvement of survival outcomes in patients with novel agents and autologous stem cell transplantation (AuSCT) [OS is 5 months prior to 2006, with increase to 12 months with the introduction of novel chemotherapy], prognosis remains poor with mortality within the first month as high as 15%.^8^

PCL is further classified as primary (pPCL) when the leukemic phase is present at diagnosis and as secondary (sPCL) when there is leukemic transformation of relapsed or refractory MM.^9^ pPCL was initially thought to represent approximately 60% of PCL cases; however, with an increase in the number of sPCL in recent years that may be attributed to prolonged survival of patients with MM, pPCL may only comprise half of cases.^10^ Patients with pPCL present at a younger age than sPCL (median age of diagnosis 55 yo vs 66 yo).

pPCL has an aggressive clinical course given its tendency to invade extramedullary sites (lymphadenopathy, hepatosplenomegaly, pleural effusion, skin, and central nervous system involvement) in up to 20% of patients.^9^, ^11^, ^12^ Higher prevalence of elevated lactate dehydrogenase (LDH) (> = 460 U/L, 48% vs 9% in MM), anemia (Hgb < 8.5 g/dL, 54% vs 31% in MM), thrombocytopenia (platelets <100 × 10^9^/L, 48% vs 9% in MM), beta‐2 microglobulin (> = 6 mg/L, 65% vs 27% in MM), hypoalbuminemia, hypercalcemia (serum calcium > = 11 mg/dL, 48% vs 20% in MM), and renal impairment (serum creatinine > = 2 mg/dL, 44% vs 21% in MM) is observed in pPCL.^11^ Additionally, osteolytic lesions are less common in pPCL (35% vs 81% of MM and 53% of sPCL).^13^ sPCL is generally even more aggressive with OS of only 1.3 to 19 months.^13^, ^14^ The median time from MM diagnosis to leukemic transformation to sPCL is approximately 20‐22 months.

Distinct immunophenotypic expression patterns are found in PCL when compared with MM. Cytogenetic abnormalities are seen in 70% of pPCL and 100% of sPCL.^15^ Multiparametric flow cytometry shows increased prevalence of CD20, CD44, CD45, CD19, and CD23 and lower CD9, CD56, CD117, and HLA‐DR.^11^, ^16^ CD56 is a neuronal cell adhesion molecule that anchors plasma cells to the bone marrow stroma, preventing their migration to extramedullary sites.^17^ Higher frequencies of t(4;14), t(11;14) and t(14;16) were observed in pPCL.^18^ Translocation involving the immunoglobulin heavy chain (IgH) locus on 14q32 is present in more than 80% on pPCL with 25%‐65% of IgH translocations in t(11;14) and is associated with leukemic transformation in MM.^19^ Elevated beta‐2 microglobulin, low serum albumin, plasma cell labeling index, elevated LDH, hypercalcemia and t(4;14) and t(14;16), partial or complete deletion of chromosome 17, deletion of 8q21, and 1p loss or lq gains have been associated with poor prognosis.^1^, ^13^

## 2 CASE PRESENTATION

An obese 76‐year‐old African American woman with sickle cell trait presented to the ED with a 1‐month history of nonproductive cough. For the past 5 days leading up to admission, she reported generalized fatigue, bilateral pulsatile tinnitus, and loose melenic stools. She had two episodes of nonbloody nonbilious vomiting on the day of presentation. She sought medical attention due to persistent loose bowel movements and worsening lethargy to the point she was unable to ambulate to the bathroom.

**Figure 1:**
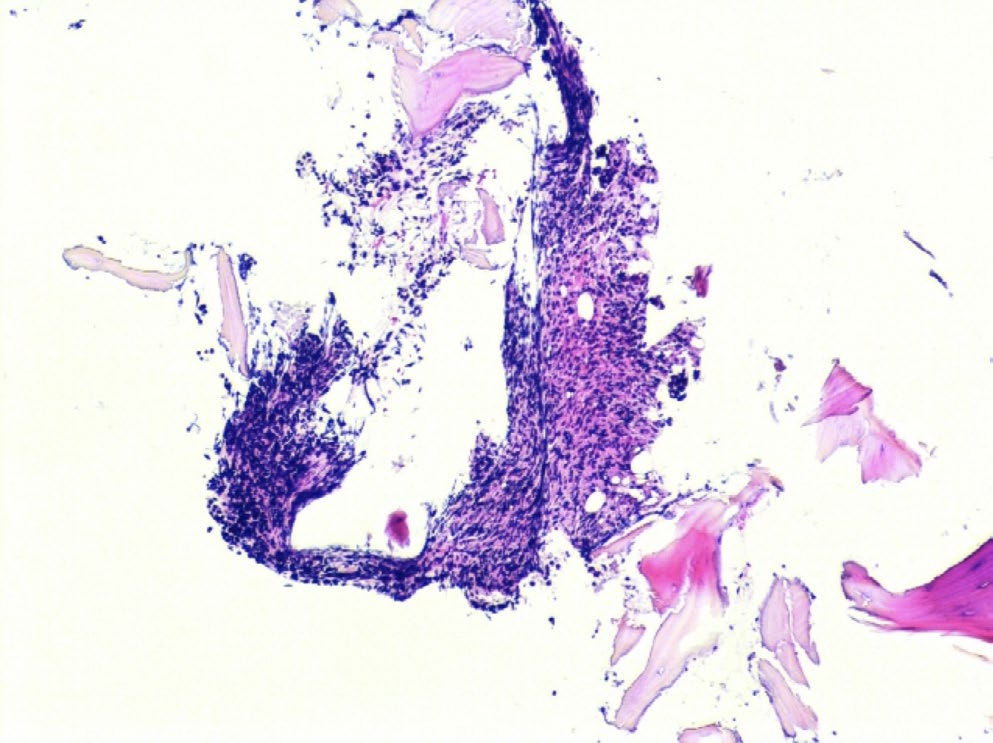
A bone marrow core biopsy specimen is minute with a small amount of hypercellular bone marrow with a cellular infiltrate displaying marked crush artifact. Rare normal bone marrow elements are identified

**Figure 2:**
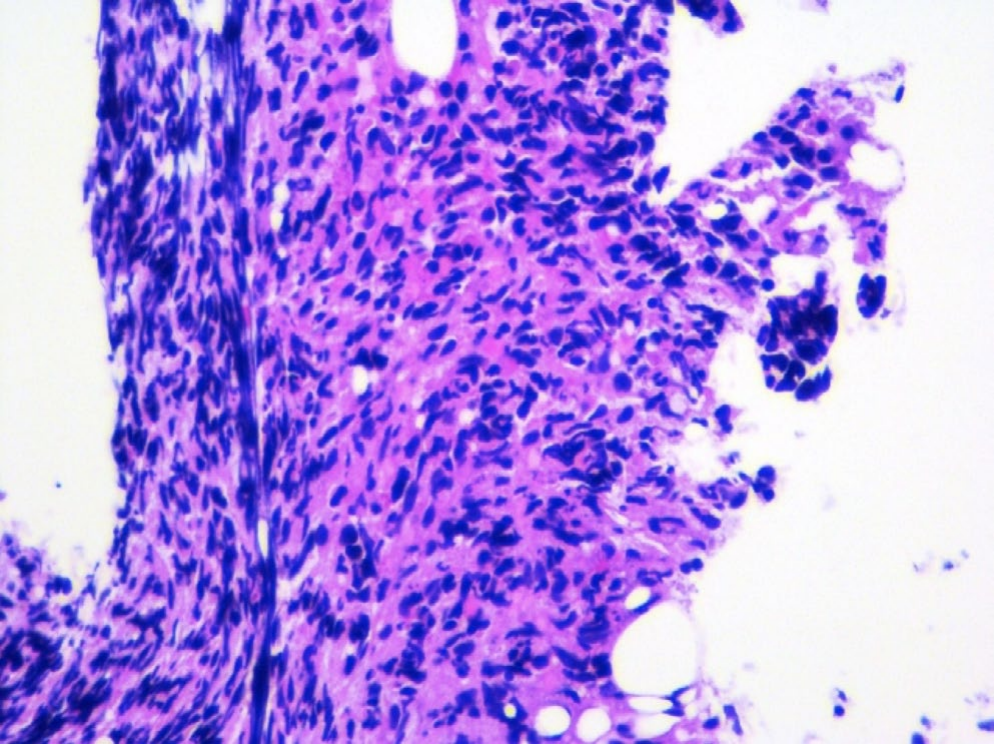
A bone marrow core biopsy specimen is minute with a small amount of hypercellular bone marrow with a cellular infiltrate displaying marked crush artifact. Rare normal bone marrow elements are identified

**Figure 3:**
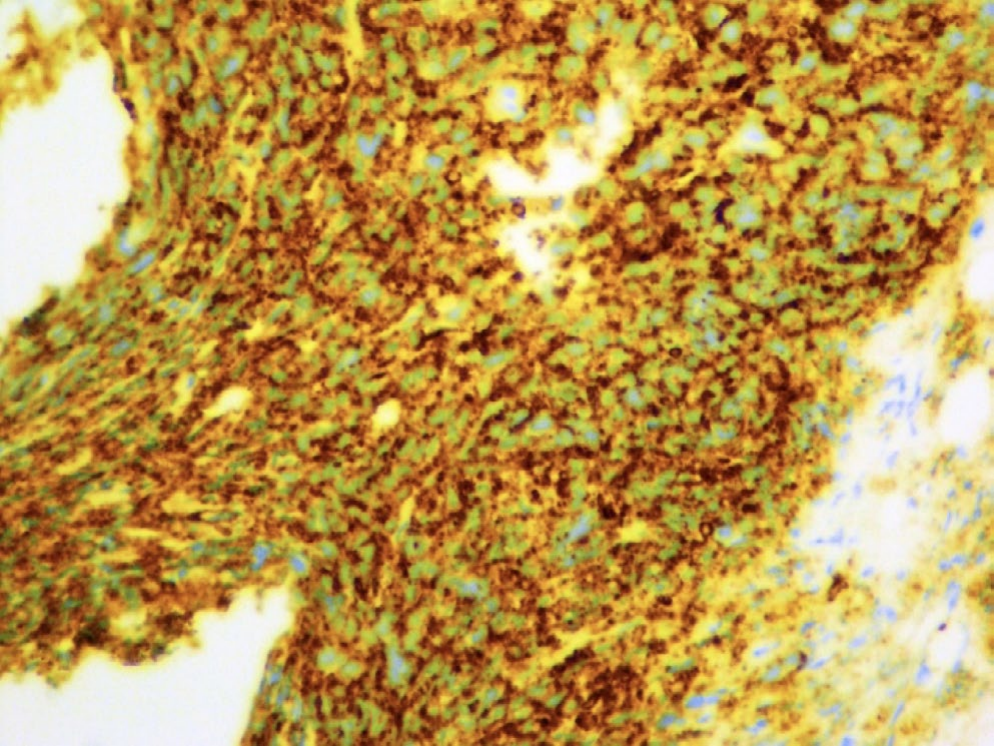
The tumor cells are strongly positive for CD138

**Figure 4:**
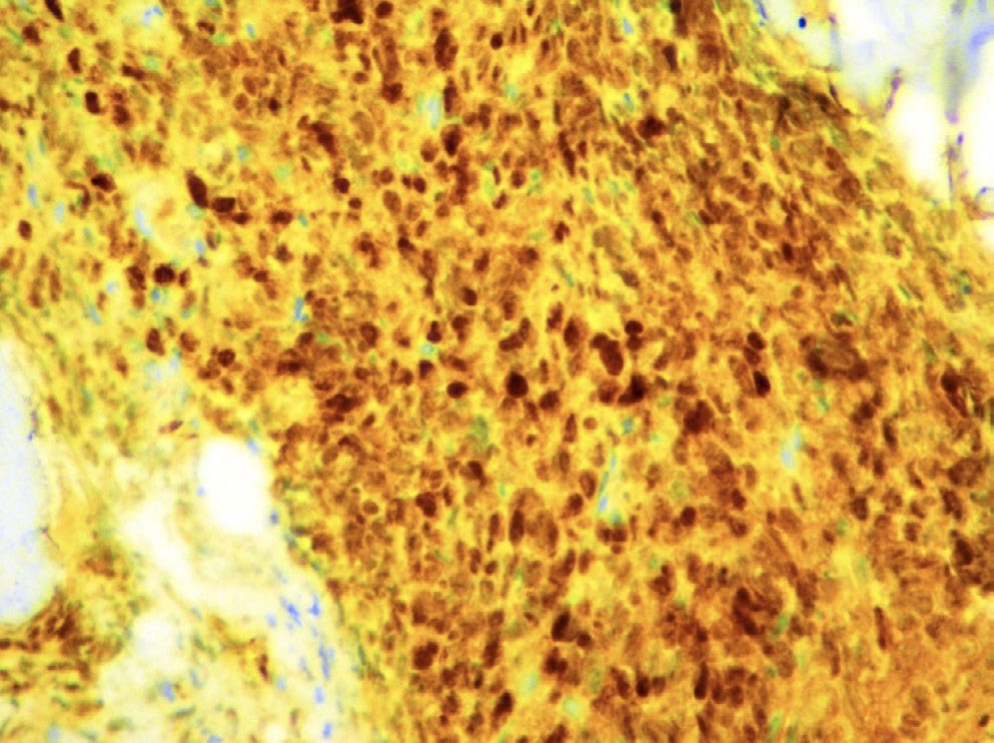
The tumor cells are strongly positive for MUM‐1

On admission, she was afebrile (97.9 F) and normotensive with 100% oxygen saturation on room air. Physical examination revealed a pallid woman of large body habitus (BMI 39) with a palpable spleen tip and bilateral lower extremity ecchymoses. Rectal examination was positive for melenic stool. She was in no acute distress, and the remainder of physical examination was unremarkable. Abdominal ultrasonography confirmed splenomegaly measuring 15.2 cm with an indeterminate 1.6 cm hypoechoic splenic lesion. Skeletal survey showed a 7 mm lucency of the parietal bone of the skull, possibly demonstrating a venous lake. Initial laboratory investigations revealed severe microcytic anemia, neutropenia, and thrombocytopenia with low reticulocyte count (Table 1). LDH was elevated. Patient was also found to have acute kidney injury on chronic kidney disease stage III with elevated creatinine. Peripheral smear revealed microcytic anemia, nucleated RBCs, target cells, thrombocytopenia, and rare schistocytes; however, it was not suggestive of a clonal B‐cell population. Initial serum protein electrophoresis (SPEP) showed one beta‐migrating paraprotein and one gamma‐migrating paraprotein with identification of 22% IgG lambda, 4% free lambda, and serum protein immunofixation electrophoresis (SIFE) IgG lambda. Urine protein electrophoresis (UPEP) revealed the presence of monoclonal protein (estimated concentration 81.8%), beta‐2 micro globulin 16, ANA: negative, IgG 2190, and uric acid 19.3. Immunophenotypic flow cytometrics studies revealed a clonal IgG lambda plasma cell population of 6% and 0.5% CD34+ myeloid blasts. Bone marrow biopsy demonstrated CD138, MUM‐1, CD20, CD117, CD3, and CD5 positive (myeloperoxidase, PAX‐5, CD56, and CD34 negative) plasma cell neoplasm (Figures 1, 2, 3, 4). Bone marrow cellularity and the extent of bone marrow involvement by plasma cells were not able to be determined on the initial biopsy due to a less than favorable biopsy specimen. Peripheral blood cell count of circulating plasma cells was not performed.

**Table 1:** Admission blood work results showing bicytopenia, elevated lactate dehydrogenase, and acute kidney injury

Hemoglobin	3.1 g/dL	Reticulocyte %	1.22%
Mean corpuscular volume	74 fL	Serum lactate dehydrogenase	223 IU/L
White blood cell	5.49 t‐h/mm^3^	Serum creatinine	3.69 mg/dL [baseline 1.6 mg/dL]
Absolute neutrophil count	1930		
Platelet	15 t‐h/mm^3^		

Due to her abrupt onset of clinical symptoms and rapid clinical cause, she was presumed to have plasma cell leukemia despite a low circulating plasma cell on flow cytometry. Due to her advanced age, she was not an ideal autologous stem cell transplant candidate. In addition, her poor clinical condition did not favor the use of VDT‐PACE, a combination of bortezomib, dexamethasone, thalidomide, cisplatin, adriamycin, cyclophosphamide, and etoposide. She received dexamethasone followed by bortezomib, cyclophosphamide, and daratumumab for 3 cycles with good response. Repeat serum protein electrophoresis (SPEP) and peripheral flow cytometry demonstrated no evidence for clonal B‐cell population, abnormal T‐cell population, or increased blast population. She was well at 6‐month follow‐up period. Provided there are no complications related to chemotherapy, she is to continue the use of bortezomib or daratumumab indefinitely at approximately 12 months as she is transplant‐ineligible. Cytogenetics testing later revealed t(11;14) q32;q23 with near triploidy and losses of chromosomes 13 and 17.

## 3 THERAPEUTIC MODALITIES

First‐line treatment of PCL is induction therapy with combinations of immune‐modulatory (IMiDs) drugs such as thalidomide and lenalidomide, proteasome inhibitors (bortezomib and carfilzomib), alkylators, or anthracyclines. Allogeneic (AlloSCT) or autologous stem cell transplantation (AuSCT) can further improve survival outcomes in eligible patients. However, optimal therapy is not yet established and overall prognosis remains poor even with treatment. Currently available novel agent‐based regimens include bortezomib with cyclophosphamide and dexamethasone (VCD), bortezomib with melphalan and prednisolone (VMP), bortezomib with doxorubicin and dexamethasone (PAD), cyclophosphamide with thalidomide and dexamethasone (CTD), thalidomide with dexamethasone (TD), or bortezomib with thalidomide and dexamethasone (VTD).^18^

Conventional chemotherapy used in the treatment of MM is no longer considered for PCL due to significantly higher early mortality rates.^18^ Conventional chemotherapy regimens include vincristine, doxorubicin and dexamethasone (VAD), melphalan and prednisone, cyclophosphamide and dexamethasone, high‐dose dexamethasone, cytarabine alone, or cyclophosphamide, doxorubicin, vincristine, and dexamethasone.^18^ In one study comparing the agents in Korean patients, progression‐free survival (PFS) and OS between those initially treated with conventional chemotherapy alone or novel agents were not statistically significant.^18^ However, median OS was significantly better in patients who were treated with novel agents with AuSCT (2.9 months in conventional therapy alone, 12.3 months in novel agents alone, 14.1 months in conventional chemotherapy with AuSCT, 31.1 months in novel agents with AuSCT, *P* < .001).^18^

Novel agents are not without their drawbacks. Dose‐dependent bortezomib‐induced peripheral neuropathy is a common cause for dose modification and drug discontinuation.^20^ Likewise, lenalidomide‐containing regimens can cause myelosuppression. Low‐dose weekly dexamethasone (40 mg) not only reduces the dose of lenalidomide from 25 mg to 15 mg but also lowers the risk of hematologic toxicities (15 to 30%), infections (20% to 8%), and thromboembolism (20% to 5%).^20^

## 4 CHEMOTHERAPEUTIC AGENTS

### 4.1 A) Immunomodulatory drugs: Thalidomide and lenalidomide

Thalidomide was one of the first IMiDs that was used for the treatment of relapsed and refractory MM. However, its efficacy in pPCL is dubious with scant reports of durable responses in small series or case reports when combined with conventional chemotherapy.^21^ Its use as a single agent did not meet any significant clinical endpoints in both pPCL and sPCL.^21^

Hyper‐CVAD regimen (cyclophosphamide‐vincristine‐doxorubicin‐dexamethasone) in combination with thalidomide showed rapid and excellent responses with prolongation of remission with AlloSCT.^22^ However, Petrucci et al observed no response to thalidomide with mortality in all 5 patients with PCL after a median of 2 months.^21^ Severe cardiac and pulmonary adverse effects are reported.^23^, ^24^

Lenalidomide, a derivative of thalidomide, is a second‐generation IMiD that has shown better efficacy in pPCL when used in a combination regimen. Patients failing thalidomide and/or bortezomib combinations have shown transient responses to lenalidomide and dexamethasone.^25^, ^26^ It is the first novel agent tested in a prospective trial in patients with pPCL.^21^

### 4.2 B) Proteasome inhibitors: Bortezomib, carfilzomib, and ixazomib

The ubiquitin‐proteasome pathway plays an important role in tumorigenesis and cell proliferation by producing substrates required for synthesis and protein modification. Bortezomib is an ubiquitin‐proteasome inhibitor with pro‐apoptotic properties that is approved for the treatment of MM.^27^ Few previous retrospective analysis have shown bortezomib‐based regimens showed efficacy with OS of about 18 to 28 months in patients with plasma cell leukemia.^28^, ^29^, ^30^, ^31^, ^32^, ^33^ In 2016, a multicenter phase 2 prospective trial that enrolled 40 patients with pPCL aged 70 years or less for four alternating cycles of bortezomib, dexamethasone plus doxorubicin or cyclophosphamide.^34^ Patients then received high‐dose melphalan followed by AuSCT.^34^ Median PFS was 15.1 months, and the OS was 36.3 months. The study concluded that patients with bortezomib combination therapy followed by transplantation induced high response rates and improved PFS.^34^

Synergism between IMiDs and proteasome inhibitors can be utilized in treatment of patients refractory to lenalidomide. This synergism can be explained by a novel mechanism of action of lenalidomide which involves increased protein ubiquitination.^35^

### 4.3 C) Bcl‐2 inhibitors: Venetoclax

The intrinsic apoptosis pathway is regulated by competing anti‐apoptotic (eg, Bcl‐2, Mcl‐1) and pro‐apoptotic proteins (eg, Bax, Bak, Bim). Cell death is induced when cellular injury causes the release of pro‐apoptotic proteins which are normally sequestered by anti‐apoptotic proteins. These pro‐apoptotic proteins translocate to the mitochondrial outer membrane and initiate a cascade that leads to increased mitochondria permeability and apoptosis.^36^ Bcl‐2 family proteins are highly associated with the survival of clonal plasma cell malignancies, whose targeted inhibition has been used in the setting of refractory disease with efficacy in those with t (11;14) abnormality.^8^ Venetoclax is a selective oral Bcl‐2‐specific BH3 mimetic that is FDA approved for the treatment of chronic lymphocytic leukemia (CLL).^8^ It has been shown in a Phase I study in those with relapsed or refractory multiple myeloma with t (11;14) abnormality to have an overall response rate (ORR) of 21% and very good partial response or better in 15%.^36^ Mcl‐1, another Bcl‐2 family protein, has been associated with resistance to Bcl‐2 inhibition when overexpressed in clonal plasma cell malignancies. Theoretically, inhibition of Mcl‐1 with bortezomib in combination with venetoclax is synergistic. A combination regimen of daratumumab‐venetoclax‐bortezomib‐dexamethasone was reported to lead to disease remission in a patient refractory to proteasome inhibitors after three cycles of therapy with repeat bone marrow biopsy showing no morphological or immunophenotypical evidence of clonal plasma cells.^8^ Dexamethasone can upregulate the expression of pro‐apoptotic molecule BIM and has been shown to have high response rates when used in combination with venetoclax and bortezomib irrespective of t (11;14) status.^36^

### 4.4 D) Anti‐CD38: Daratumumab

Natural killer (NK) cell‐based immunotherapy is a therapeutic approach to refractory MM. Daratumumab, an engineered monoclonal antibody (IgG1k), binds to CD38 surface antigen that is overexpressed in MM cells. This leads to activation of NK antibody‐dependent cellular cytotoxicity (ADCC) activity.^37^ However, their antitumor effects seem dependent on CD38 expression since ADCC activity was largely absent in CD38 low or negative cells.^38^ Daratumumab can exert toxic effect on healthy cells, leading to decreased number of NK cells. Termed fratricide, a phenomenon whereby binding of the mAb against CD38+ NK cells leads to ADCC activation against other CD38+ NK cells bound to daratumumab.^38^ It is approved by the Food and Drug Administration (FDA) initially in 2015 as a second‐line agent in the treatment of MM in patients who have received at least three prior therapies. The following year, the FDA further expanded the use of daratumumab in combination with lenalidomide or bortezomib and dexamethasone in those who have received at least one prior therapy. Beginning in May 2018, it can be used in combination with bortezomib, melphalan, and prednisone for treatment‐naive MM who are transplant‐ineligible. There are currently no published trials that demonstrate its efficacy in pPCL. There is currently one case report that showed a rapid and deep response in a pPCL with t(11;14).^39^

## 5 DISCUSSION

Plasma cell leukemia is a rare blood dyscrasia with an aggressive clinical course and dismal prognosis. Due to the rarity and fulminant nature of the condition, there are limited data driving dialogue for optimal treatment strategies. Likely, future studies will involve collaborative efforts between institutions to elucidate the disease entity. There is a need for an effective early treatment with a durable therapeutic response. Recent advances in chemotherapeutic regimens particularly with the introduction of novel agents in combination with stem cell transplant have led to improved rate, quality of response, and median OS in patients with PCL.

There remains a need for a more comprehensive diagnostic criterion. The current diagnostic definition of PCL is not prospectively studied and delays many from receiving early aggressive management strategies due to underdiagnosis. The presence of 5% circulating clonal plasma cells has been suggested to improve detection.^40^ This has been echoed by a recent Mayo Clinic study in December 2018 that showed those diagnosed between 1971 and 2016 with > = 5% circulating plasma cells (CPCs) had much poorer survival outcomes compared with those who did not have CPCs on diagnosis.^41^ The successful treatment of this patient with VCD and daratumumab may prove an effective induction strategy for similar patients with an aggressive presentation, who are not candidates for AuSCT. This is the second case report to report the use of daratumumab in plasma cell leukemia. More research is needed in exploring genomic variations which may potentially identify various novel biomarkers that may serve as prognostic markers. Overall clinical course remains poor, signaling a need for more agents, and effective management strategies.

## CONFLICT OF INTEREST

None declared.

## AUTHOR CONTRIBUTION

Sam Ngu MD: Lead author of the manuscript. Divya Asti MBBS: made considerable contributions to the manuscript as a writer and expanded on treatment modalities. Gautam Valecha MBBS and Nishitha Thumalapally MBBS: played supportive roles as consulting oncologists who offered clinical insights and helped to revise the paper. Manisha Pant MBBS: played a supportive role as a consulting physician who helped to revise the paper. Alexander Bershadskiy MD: Lead oncologist who made the clinical diagnosis  and developed the treatment course for the patient. He oversaw all aspects of patient care and was also deeply invested in the revision process of this article.

## References

[1] Dimopoulos MA , Palumbo A , Delasalle KB , Alexanian R . Primary plasma cell leukaemia. Br J Haematol. 1994;88:754‐759. 7819100 10.1111/j.1365-2141.1994.tb05114.x

[2] Kyle RA , Maldonado JE , Bayrd ED . Plasma cell leukemia. Report on 17 cases. Arch Intern Med. 1974;133:813‐818. 4821776 10.1001/archinte.133.5.813

[3] Shah R , Mohite S , Baladandayuthapani V , et al. Circulating plasma cells by routine complete blood count identify patients with similar outcome as plasma cell leukemia. Blood. 2013;122(21):5356.

[4] An G , Qin X , Acharya C , et al. Multiple myeloma patients with low proportion of circulating plasma cells had similar survival with primary plasma cell leukemia patients. Ann Hematol. 2014;94:257‐264. 25231928 10.1007/s00277-014-2211-0

[5] Gonsalves WI , Morice WG , Rajkumar V , et al. Quantification of clonal circulating plasma cells in relapsed multiple myeloma. Br J Haematol. 2014;167:500‐505. 25113422 10.1111/bjh.13067 PMC4211997

[6] Neri A , Todoerti K , Lionetti M , et al. Primary plasma cell leukemia 2.0: advances in biology and clinical management. Expert Rev Hematol. 2016;9(11):1063‐1073. 27759436 10.1080/17474086.2016.1244002

[7] Ramsingh G , Mehan P , Luo J , Vij R , Morgensztern D . Primary plasma cell leukemia: a surveillance, epidemiology, and end results database analysis between 1973 and 2004. Cancer. 2009;115:5734‐5739. 19877113 10.1002/cncr.24700

[8] Gonsalves WI , Rajkumar SV , Go RS , et al. Trends in survival of patients with primary plasma cell leukemia: a population‐based analysis. Blood. 2014;124:907‐912. 24957143 10.1182/blood-2014-03-565051 PMC4126330

[9] Mina R , D’Agostino M , Cerrato C , Gay F , Palumbo A . Plasma cell leukemia: update on biology and therapy. Leuk Lymphoma. 2016;58(7):1538‐1547. 27819179 10.1080/10428194.2016.1250263

[10] Fernández de Larrea C , Kyle RA , Durie B , et al. Plasma cell leukemia: consensus statement on diagnostic requirements, response criteria and treatment recommendations by the International Myeloma Working Group. Leukemia. 2013;27:780‐791. 23288300 10.1038/leu.2012.336 PMC4112539

[11] Garcia‐Sanz R , Orfao A , González , et al. Primary plasma cell leukemia: clinical, immunophenotypic, DNA ploidy, and cytogenetic characteristics. Blood. 1999;93:1032‐1037. 9920853

[12] Blade J , Kyle RA . Nonsecretory myeloma, immunoglobulin D myeloma, and plasma cell leukemia. Hematol Oncol Clin North Am. 1999;13:1259‐1272. 10626149 10.1016/s0889-8588(05)70125-8

[13] Tiedemann RE , Gonzalez‐Paz N , Kyle RA , et al. Genetic aberrations and survival in plasma cell leukemia. Leukemia. 2008;22(5):1044‐1052. 18216867 10.1038/leu.2008.4 PMC3893817

[14] Sekiguchi Y , Shimada A , Wakabayashi M , et al. A case of secondary plasma cell leukemia resistant to novel agents, in which stringent complete remission was achieved and maintained for a long period of time after VAD therapy and tandem autologous transplantation. Int J Clin Exp Pathol. 2014;7(9):6313‐6322. 25337285 PMC4203256

[15] Fonseca R , Barlogie B , Bataille R , et al. Genetics and cytogenetics of multiple myeloma: a workshop report. Cancer Res. 2004;64:1546‐1558. 14989251 10.1158/0008-5472.can-03-2876

[16] Kraj M , Kopec‐Szelzak J , Poglod R , Kruk B . Flow cytometric immunophenotypic characteristics of plasma cell leukemia. Folio Histochem Cytobiol. 2011;9(1):168‐182. 10.5603/fhc.2011.0024 21526505

[17] Jelinek T , Kryuov F , Rihova L , Hajek R . Plasma cell leukemia: from biology to treatment. Eur J Haematol. 2015;95:16‐26. 25778450 10.1111/ejh.12533

[18] Hong J , Lee JH . Recent advances in multiple myeloma: a Korean perspective. Korean J Intern Med. 2016;31(5):820‐834. 27604794 10.3904/kjim.2015.408 PMC5016289

[19] Pratt G . Molecular aspects of multiple myeloma. Mol Pathol. 2002;55(5):273‐283. 12354927 10.1136/mp.55.5.273 PMC1187254

[20] Ueda S , Kubo M , Matsuura N , et al. Stringent complete remission of primary plasma cell leukemia with reduced‐dose bortezomib, lenalidomide and dexamethasone: a case report and review of the literature. Intern Med. 2013;52:1235‐1238. 23728562 10.2169/internalmedicine.52.0001

[21] Petrucci MT , Martini V , Levi A , et al. Thalidomide does not modify the prognosis of plasma cell leukemia patients: experience of a single center. Leuk Lymphoma. 2007;48:180‐182. 17325863 10.1080/10428190601007570

[22] Murthy V , Mwirigi A , Ward S , Rassam S . Rapid and excellent response to hyper‐CVAD, particularly with thalidomide, in plasma cell leukaemia and long term remissions following allogeneic stem cell transplantation. Blood. 2009;114:4263.

[23] Ballanti S , Mastrodicasa E , Bolli N , et al. Sustained ventricular tachycardia in a thalidomide‐treated patient with primary plasma‐cell leukemia. Nat Clin Pract Oncol. 2007;4:722‐725. 18037876 10.1038/ncponc1008

[24] Pretz J , Medeiros BC . Thalidomide‐induced pneumonitis in a patient with plasma cell leukemia: no recurrence with subsequent lenalidomide therapy. Am J Hematol. 2009;84:698‐699. 19691102 10.1002/ajh.21495

[25] Musto P , Pietrantuono G , Guariglia R , et al. Salvage therapy with lenalidomide and dexamethasone in relapsed primary plasma cell leukemia. Leuk Res. 2008;32:1637‐1638. 18433866 10.1016/j.leukres.2008.03.013

[26] Guglielmelli T , Merlini R , Giugliano E , Saglio G . Lenalidomide, melphalan, and prednisone association is an effective salvage therapy in relapsed plasma cell leukaemia. J Oncol. 2009;2009:1‐2. 10.1155/2009/867380 PMC2786165 20011654

[27] Reimold AM , Iwakoshi NN , Manis J , et al. Plasma cell differentiation requires the transcription factor XBP‐1. Nature. 2001;412:300‐307. 11460154 10.1038/35085509

[28] Esparís‐Ogando A , Alegre A , Aguado B , et al. Bortezomib is an efficient agent in plasma cell leu‐ kemias. Int J Cancer. 2005;114:665‐667. 15609327 10.1002/ijc.20793

[29] Musto P , Rossini F , Gay F , et al. Efficacy and safety of bortezomib in patients with plasma cell leukemia. Cancer. 2007;109:2285‐2290. 17469169 10.1002/cncr.22700

[30] van de Donk N , Lokhorst HM , Anderson KC , Richardson PG . How I treat plasma cell leukemia. Blood. 2012;120:2376‐2389. 22837533 10.1182/blood-2012-05-408682 PMC3757364

[31] D'Arena G , Valentini CG , Pietrantuono G , et al. Frontline chemotherapy with bortezomib‐containing combinations improves response rate and survival in primary plasma cell leukemia: a retrospective study from GIMEMA Multiple Myeloma Working Party. Ann Oncol. 2012;23:1499‐1502. 22039089 10.1093/annonc/mdr480

[32] Lebovic D , Zhang L , Alsina M , et al. Clinical outcomes of patients with plasma cell leukemia in the era of novel therapies and hematopoietic stem cell transplantation strategies: a single‐institution experience. Clin Lymphoma Myeloma Leuk. 2011;11:507‐511. 21813352 10.1016/j.clml.2011.06.010

[33] Al‐Nawakil C , Tamburini J , Bardet V , et al. Bortezomib, doxorubicin and dexamethasone association is an effective option for plasma cell leukemia induction therapy. Leuk Lymphoma. 2008;49:2012‐2014. 18720213 10.1080/10428190802290660

[34] Royer B , Minvielle S , Diouf M , et al. Bortezomib, doxorubicin, cyclophosphamide, dexamethasone induction followed by stem cell transplantation for primary plasma cell leukemia: a prospective phase II study of the Intergroupe Francophone du Myelome. J Clin Oncol. 2016;34(18):2125‐2132. 27114594 10.1200/JCO.2015.63.1929

[35] Fink EC , Ebert BL . The novel mechanism of lenalidomide activity. Blood. 2015;126(21):2366‐2369. 26438514 10.1182/blood-2015-07-567958 PMC4653765

[36] Kumar S , Kaufman JL , Gasparetto C , et al. Efficacy of venetoclax as targeted therapy for relapsed/refractory t(11;14) multiple myeloma. Blood. 2017;130(22):2401‐2409. 29018077 10.1182/blood-2017-06-788786

[37] Nijhof IS , van Bueren J , van Kessel B , et al. Daratumumab‐mediated lysis of primary multiple myeloma cells is enhanced in combination with the human anti‐KIR antibody IPH2102 and lenalidomide. Haematologica. 2015;100(2):263‐268. 25510242 10.3324/haematol.2014.117531 PMC4803142

[38] Mahaweni NM , Bos G , Mitsiades CS , et al. Daratumumab augments alloreactive natural killer cell cytotoxicity towards CD38+ multiple myeloma cell lines in a biochemical context mimicking tumour microenvironment conditions. Cancer Immunol Immunother. 2018;67:861. 29500635 10.1007/s00262-018-2140-1 PMC5951903

[39] Gonsalves WI , Buadi FK , Kumar SK . Combination therapy incorporating Bcl‐2 inhibition with Venetoclax for the treatment of refractory primary plasma cell leukemia with t (11;14). Eur J Haematol. 2018;100:215‐217. 29064593 10.1111/ejh.12986

[40] Granell M , Calvo X , Garcia‐Guiñón A , et al. Prognostic impact of circulating plasma cells in patients with multiple myeloma: implications for plasma cell leukemia definition. Haematologica. 2017;102(6):1099‐1104. 28255016 10.3324/haematol.2016.158303 PMC5451342

[41] Ravi P , Kumar SK , Roeker L , et al. Revised diagnostic criteria for plasma cell leukemia: results of a Mayo Clinic study with comparison of outcomes to multiple myeloma. Blood Cancer J. 2018;8(12):116. 30442928 10.1038/s41408-018-0140-1 PMC6238010

